# Simultaneous UHPLC-UV Determination of Hericenones, Hericenes, Erinacines and Ergosterol in *Hericium erinaceus* Raw Materials or Products

**DOI:** 10.3390/molecules31030569

**Published:** 2026-02-06

**Authors:** Yijin Tang, Ozan Kahraman, Anthony J. Goos, Christine Fields

**Affiliations:** Applied Food Sciences, Inc., 2500 Crosspark Road, Coralville, IA 52241, USA; ozan@appliedfoods.com (O.K.); ajgoos@appliedfoods.com (A.J.G.)

**Keywords:** *Hericium erinaceus* (*H. erinaceus*), UHPLC-UV, hericenone, hericene, erinacine, ergosterol, single-laboratory method validation (SLMV), chemical markers, quality control

## Abstract

This study describes a single-laboratory validation of an ultra-high-performance liquid chromatographic (UHPLC) method for the determination of key compounds like hericenones, hericenes, erinacines, and ergosterol in *Hericium erinaceus* (*H. erinaceus*, Lion’s Mane) raw materials and finished products. The expanding market for *Hericium erinaceus* (Lion’s Mane) has increased the need for practical, routine-ready analytical methods that can quantify characteristic marker compounds and strengthen quality control across both raw materials and finished products. In this study, an ultra-high-performance liquid chromatographic (UHPLC) separation method was developed for the determination of hericenones, hericenes, erinacines, and ergosterol in *Hericium erinaceus* raw materials and finished products. Under the optimized conditions, the major target analytes—hericenones, hericenes, erinacine A, and ergosterol—were fully resolved (R_s_ > 1.5) within 38 min using an HSS T3 column at 30 °C. All the peaks in the LC chromatogram of *Hericium erinaceus* samples and standard solutions were structurally confirmed by LC–UV-MS/MS based on the possible mass spectra. The quantitative calibration curves were linear, covering a range of 10–300 μg/mL for hericenone C, D and E, and hericene A, D and C; 3–100 μg/mL for deacylhericenone and deacylhericene; 1–50 μg/mL for erinacine A, and 5–200 μg/mL for ergosterol. Limits of quantification (LOQs) for hericenone C, D, and E and for hericene A, D, and C were approximately 9.263, 4.545, 4.650, 1.854, 10.72, and 11.18 µg/mL, respectively, while LOQs for deacylhericenone and deacylhericene were 1.083 and 2.109 µg/mL. Erinacine A and ergosterol showed LOQs of 0.642 and 8.352 µg/mL, respectively. The recovery of ergosterol was evaluated for the method at two different levels: 91.6~93.9% for 0.2% spiking and 93.0~102.6% for 0.08% spiking. The method was successfully validated, demonstrating inter-day Relative Standard Deviation (RSD) values between 1.1% and 5.7% for detected analytes across diverse matrices. This validated method provides a consistent quantification of hericenones, hericenes, erinacine A, and ergosterol across a range of commercial products and raw *Hericium erinaceus* materials, providing a sensitive and reliable tool for product characterization and quality control. This method provides QC laboratories with a robust, UV-based tool for standardized product characterization without requiring mass spectrometry.

## 1. Introduction

*Hericium erinaceus* (*H. erinaceus,* also known as Lion’s mane mushroom) is a valuable edible and medicinal mushroom [1]. *H. erinaceus* has attracted much attention due to its potential neuroprotective [2,3,4,5] and cognitive-enhancing functions [6,7,8]. Many studies suggested these effects were biologically plausible and may involve several related mechanisms, such as support of neurotrophic signaling, reduced oxidative stress, and modulation of neuroinflammation. However, it is also emphasized that consistent results in human studies depend strongly on using well-identified and standardized materials, along with robust analytical methods to ensure product quality across the supply chain.

**Figure 1:**
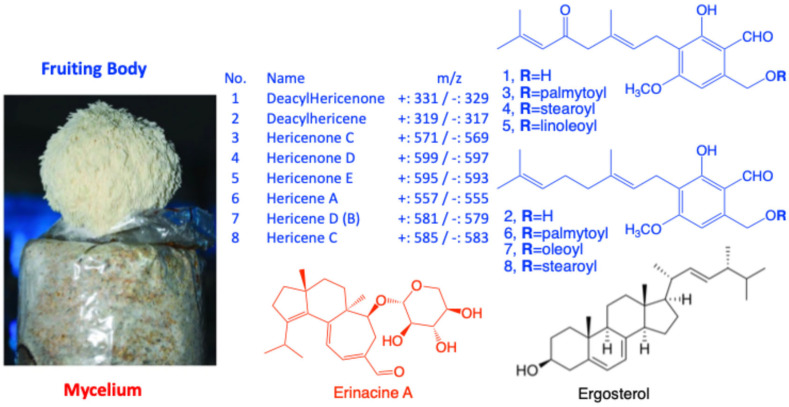
Fruit body and mycelium of *H. erinaceus* and chemical structures of major hericenones and hericenes, erinacine A and ergosterol.

A key characteristic of *H. erinaceus* is that it contains a range of chemically distinct marker metabolites, and these are not evenly distributed across the mushroom [1]. In general, the fruiting body is richer in aromatic, mostly lipophilic secondary metabolites—most notably hericenones and hericenes, e.g. [9,10,11,12,13]—whereas the mycelium is more strongly associated with erinacines (cyathane-type diterpenoids) [2,8,14,15]. These compounds are often linked to the prevention and treatment of neurodegenerative diseases because they can induce the synthesis and secretion of nerve growth factor (NGF). In addition, ergosterol (a precursor to vitamin D2) is a naturally occurring sterol found in many mushrooms, including *H. erinaceus*, contributing to mushroom health-promoting properties [16,17]. Due to it being widely present in mushroom tissues, ergosterol is commonly used as a compositional marker for mushroom-derived materials. Therefore, accurate and simultaneous measurement of the major hericenones, hericenes, ergosterol and erinacines in *H. erinaceus* materials (Figure 1) is essential for reliable quality control and for supporting the efficacy of the rapidly expanding range of *H. erinaceus* health products.

Reversed-phase high-performance liquid chromatography (RP-HPLC)–Ultraviolet–Visible (UV) or –mass spectra (MS) have been reported for detecting or monitoring certain key compounds present in *H. erinaceus* materials [18,19,20,21]. The LC-UV or -MS was considered an effective method with good separation and high accuracy among many existing analytical methods; however, no reported LC-UV or -MS method has been fully developed and validated for determining those key compounds in *H. erinaceus* materials. There are two major challenges for developing the quantitative analytical method of those compounds in *H. erinaceus* materials: the availability of standards (even very expensive) and enough separation of those compounds in the complicated matrices by LC. The objective of this study was to develop and validate a single-laboratory UHPLC-UV method, based on AOAC and ICH Q2(R1) guidelines [22,23], for the simultaneous quantification of major bioactive hericenones, hericenes, ergosterol, and erinacine A using commonly available instrumentation to obtain reliable simultaneous quantification of the major bioactive hericenones, hericenes, ergosterol, and erinacines in *H. erinaceus* raw materials, mycelia or derived products.

## 2. Results and Discussions

### 2.1. Identification of Hericenones, Hericenes, Ergosterol and Erinacines with Sample Preparation

**Figure 2:**
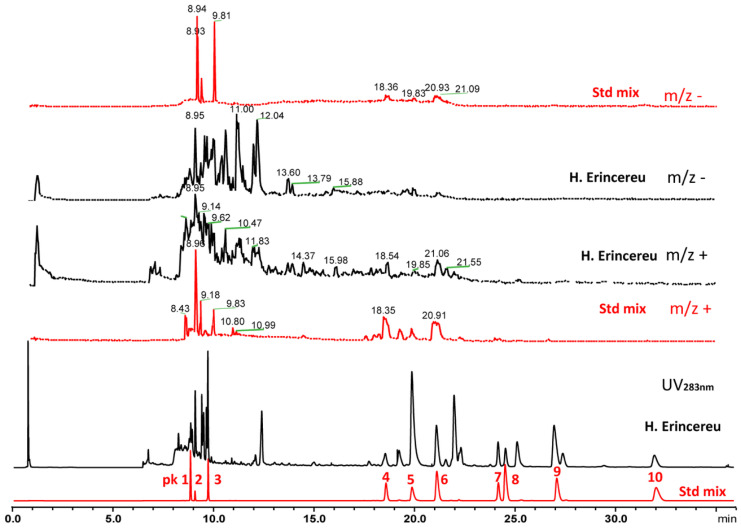
UHPLC-UV-MS chromatography of mixed standards (hericenones, hericenes, ergosterol and erinacines) and an *H. erinaceus* sample (mixed ethanol extract from fruit body and mycelia). Peak 1~10: deacylhericenone, erinacine A, deacylhericene, hericenone E, ergosterol, hericenone C, hericenone D, hericene D, hericene A and hericene C.

Available reference standards and *H. erinaceus* samples were analyzed during method development using UHPLC–UV and MS detection. UV chromatograms were recorded at 283 nm, and MS data were monitored using the total ion chromatogram (TIC) (Figure 2).

The UHPLC–UV results showed that the major marker compounds exhibited characteristic absorbance wavelengths, with hericenones and hericenes showing strong absorption at 292 nm, ergosterol at 283 nm, and erinacines at 340 nm [18,24,25,26]. The UV spectra of the reference standards are provided in Supplementary Materials Figure S1. Compound identities for the hericenones, hericenes, and erinacines were further confirmed based on their mass spectra (Supplementary Materials Figure S2). However, the mass spectra show that only a few bioactive compounds had good ionization and most of the major hericenones, hericenes and erinacines are not well-responsive as either positive or negative MS spectra; in particular, ergosterol exhibited poor ionization in electrospray mode, and significant ion suppression was observed for hericenones and hericenes compared to stable UV responses. LC–MS is highly effective for confirming compound identity and supporting structural characterization in *H. erinaceus* matrices. However, for routine quantitation, its performance can be constrained by low and variable ionization efficiencies and substantial matrix effects in many *H. erinaceus* samples. For marker compounds present at non-trace levels, LC–UV/PDA (DAD) is often better suited for day-to-day quantitative testing because it provides robust precision and a faster analytical workflow. Given these considerations, LC–UV is expected to deliver good repeatability, typically with a relative standard deviation of about 2–5% across concentrations from ~10 ppm to 1%, and generally not exceeding 10% at lower concentration levels [22]. Quantification for this method was chosen at 283 nm for all analytes due to the various matrices and the ability to quantify all compounds at that UV wavelength. This allows a QC lab to use one wavelength to quantify the hericenones, hericenes, ergosterol, and erinacine all at one time, rather than quantifying each analyte individually at different wavelengths.

### 2.2. Method Validation

#### 2.2.1. Specificity/Resolution

(U)HPLC-UV is a more applicable method for the quantitation of hericenones, hericenes, ergosterol and erinacine compounds in most *H. erinaceus* raw materials or products. *H. erinaceus* raw material was used for method development, which started with gradient conditions and column screening (Supplementary Materials Figure S3). More than 10 different hericenones or hericenes were observed in the LC of the *H. erinaceus* sample. In order to obtain good separations in the ranges of 8~10 min and 17~28 min (covering most hericenones and hericenes), the best LC (Figure 2) for hericenones, hericenes, ergosterol and erinacines was achieved using HSS T3 with the gradient conditions listed in Table 1.

Each individual reference standard was injected into the UHPLC-UV to compare with the standard mixture. Identification of hericenones, hericenes, ergosterol and erinacine compounds in the test materials was determined by comparing peak retention times and UV spectra (Supplementary Materials Figure S1) to the reference standards. Representative chromatograms of the standard mixture and mixed *H. erinaceus* materials with fruit body and mycelia are displayed in Figure 2. Under the chromatographic conditions used in the present study, all the major hericenones, hericenes, ergosterol and erinacine were eluted separately following this order: deacylhericenone, erinacine A, deacylhericene, hericenone E, ergosterol, hericenone C, hericenone D, hericene D, hericene A and hericene C. Values for the relative retention times (α), retention factors (*k*′) and chromatographic resolutions (R_s_), calculated from the LC analysis of the *H. erinaceus* standard mixtures (Figure 2), are summarized in Table 2. Retention factors (*k*′) were calculated as *k*′ = (*t_r_* − *t*_0_)/*t*_0_, where *t_r_* is the retention time of the analyte and *t*_0_ is the retention time of unretained compounds (solvent front, determined as 0.85 min using the first baseline disturbance). The *k*′ values were within the optimum range (*k*′ > 2) for satisfactory chromatographic elution. Excellent chromatographic specificity was observed with good resolution of the peaks (R_s_ > 1.5) and no significant interfering peaks for all compounds in the mixed standard sample. The total chromatography run time was 38.5 min. Although the 38.5 min runtime is longer than some UHPLC separations, it represents a necessary compromise to ensure a resolution (R_s_ > 1.5) for the complex 10-analyte mixture.

#### 2.2.2. Standard Linearity

Linearity was assessed using mixed calibration standards prepared at the target concentration levels as described above. Each peak area of the chromatograms was recorded as the UV response at 283 nm for all the hericenones, hericenes, ergosterol and erinacines. Calibration curves were constructed by plotting peak area vs. the concentration of the standard compounds (Supplementary Materials Figure S4). Regression analyses were performed using Lab Solution software (ver. 5.87). Calibration curves were linear across the evaluated concentration range, with coefficients of determination (R^2^) greater than 0.999. The normalized intercept/slope of the regression line and the coefficient of correlation were calculated for the whole data set. The method was evaluated based on the coefficient of linearity and intercept values, as summarized in Table 3.

#### 2.2.3. Limit of Detection and Limit of Quantification

The limit of detection (LOD) and limit of quantification (LOQ) of the hericenones, hericenes, ergosterol and erinacine standard assay was determined by the standard deviation of y-intercepts over the slope of the regression lines from three replicated calibration curves on different days [23]. The LODs and LOQs for the major hericenones, hericenes, ergosterol and erinacine were calculated and summarized in Table 3.

#### 2.2.4. Recovery

A spike recovery experiment was performed to validate the extraction efficiency and overall method accuracy. Considering the prohibitive cost and limited commercial availability of certified reference materials for all ten unique compounds, a representative spike recovery was performed using ergosterol. Spike recovery experiments were performed by spiking ergosterol into the post-extraction pomace from *H. erinaceus* raw material at two levels: medium (0.2%) and low (0.08%) (Table 4). The average recoveries for ergosterol were found to be within the acceptable ranges of 92% to 105% at the 0.2% spiking concentration and 90% to 108% at the 0.08% spiking concentration. Specifically, recovery was 92.65 ± 1.19% at 0.2% and 97.67 ± 4.63% at 0.08%. These results suggest excellent extraction efficiency and reliable overall method performance. The slightly higher recovery at the lower spike (0.08%) level may reflect the optimized extraction conditions (3 × 3 mL solvent per 0.5 g sample), which were selected to improve recovery of highly hydrophobic analytes such as ergosterol, some hericenones and hericenes at the lower range. Regarding the sample preparation, 1×, 2× and 3× extractions were performed on one of the products (FFP5). The result (Supplementary Materials Table S1) indicated that 3× extractions did provide higher yields, especially for the high-level (close to or more than 1 mg/g) compounds, such as ergosterol (~1.798 mg/g) with relative 87.61% yield for 1× and 91.48% yield for 2×, and hericene A (~0.985 mg/g) with relative 92.46% yield for 1× and 96.51% yield for 2×, while the relative yields were compared to 3× extraction. Therefore, 3× extractions were performed for the sample preparations of all raw materials or products in this study.

#### 2.2.5. Precision

The precision and accuracy of the method were assessed by determining the intraday precisions (n = 5) from repeating the analysis of the *H. erinaceus* samples on the same day and the interday precisions (n = 3 × 5, overall 15) from analyzing the same *H. erinaceus* samples over different days. Both intraday and inter-day precisions were calculated as RSD_r_ (%) = (standard deviation)/(mean) × 100. As summarized in Table 5, all ten analytes showed adequate precision in the tested solid matrices across the concentration levels where they were detectable.

Several *H. erinaceus* raw materials or finished commercial products, including AFS finished products (AFP), were analyzed using a quick and effective LC screening. Target markers (hericenones, hericenes, ergosterol, and erinacines) were analyzed after preparing a concentrated ethanol extract corresponding to approximately 500 mg of *H. erinaceus* raw material per ~1 mL (and not exceeding 2 mL) of ethanol. The LC screening results (Supplementary Materials Figure S5) showed that, in some *H. erinaceus* raw materials and finished products, most of the target bioactive compounds were below the method’s LOD or LOQ. Full LC quantitation of hericenones, hericenes, ergosterol, and erinacine A was therefore performed only for samples in which these compounds were detectable, and the results are summarized in Table 6.

Ergosterol was consistently detected in both *H. erinaceus* fruiting body and mycelium materials, with concentrations ranging from 0.5 to 2 mg/g, and the level of ergosterol is variable depending on the mushroom species, how the material is processed, and how the sample is prepared for testing [24,27]. Only *H. erinaceus* mycelia were reported to contain erinacines [8,14,15], and erinacine A was detected at about 0.2 mg/g for the MFP-01 sample in this study (Table 5), while this sample contained very low levels of hericenones or hericenes. In contrast, for fruiting-body samples (FFP), hericenones and hericenes were detected at a higher level, and erinacine A was not detected. Across the fruiting-body materials evaluated, their concentrations varied widely, ranging from 0.005 to 1 mg/g (Table 5 and Table 6). In some materials, target compounds were present at very low levels (near or below the LOQ), which contributed to higher variability, with RSD values of approximately 6–12%. The concentrations of hericenones and hericenes were strongly influenced by the quality of the *H. erinaceus* fruiting-body raw material, including factors such as strain differences [10,21,28] and harvest stage [19]. Therefore, routine monitoring of fruiting-body quality using hericenones and hericenes as marker compounds is important for consistent product characterization and quality control. The frequent observation of markers below the LOQ (BLQ) in commercial products highlights significant variability in the market and the urgent need for standardized quantification

## 3. Materials and Methods

### 3.1. Chemicals and Materials

Acetonitrile (ACN, HPLC grade), formic acid (FA, LC–MS grade), water (H_2_O, LC–MS grade) and methanol (MeOH, LC–MS grade) were purchased from Fisher Scientific (Waltham, MA, USA). Reference standard compounds of Hericene C (98%), Hericene A (98%), Hericene D (95%), Hericenone C (98%), Hericenone E (90%), DeacylHericene (98%) and DeacylHericenone (98%) were purchased from Biopurify Phytochemicals (Chengdu, Sichuan, China). Standard compounds of Hericenone D (98%) and Erinacine A (93.8%) were the products from ChromaDex (Longmont, CO, USA). Ergosterol was from TCI Americas (Portland, OR, USA) and purchased from Fisher Scientific (Waltham, MA, USA). Ultrapure (18 MΩ) water was produced using a Barnstead^TM^ GenPure^TM^ Pro Water Purification System from Thermo Scientific (Waltham, MA, USA).

*H. erinaceus* fruiting body and/or mycelial raw materials (RW) were sourced from Applied Food Sciences, Inc. (AFS, Kerrville, TX, USA) and AFS-collaborated companies. Commercial *H. erinaceus* finished products (powders and dry-filled capsules, FP) were purchased from local retail food markets (Coralville, IA, USA).

### 3.2. Instrumentation

Method development and validation studies were performed on a Shimadzu Nexera-X2 UHPLC system (Shimadzu Scientific Instruments, Columbia, MD, USA), equipped with an LC-30AD pump, a SIL-30AC autosampler with a thermostated unit, a thermostated column compartment, and an SPD-M30A PDA detector. The UHPLC system was also interfaced with a tandem Q-Exactive Orbitrap mass spectrometer (Thermo Fisher Scientific Inc., San Jose, CA, USA). High-resolution MS and MS^2^ spectra were obtained on the Q-Exactive Orbitrap mass spectrometer equipped with a heated electrospray ionization source operated in both positive and negative ion modes. The optimized parameters were set as follows: capillary voltage, 3.0 kV; sheath gas flow rate, 35 arbitrary unit; auxiliary gas flow rate, 5 arbitrary unit; sweep gas flow rate, 5 arbitrary unit; capillary temperature, 325 °C; sheath gas heater temperature, 200 °C. MS scans were recorded in a mass range of *m*/*z* 100–1500 at a resolution of 70,000 with an AGC target of 3 × 10^6^. After each MS scan, up to 5 of the most abundant multiply charged ions were selected for fragmentation. MS^2^ scans were recorded in a mass range of *m*/*z* 50 to the parent ion at a resolution of 17,500 with an AGC target of 1 × 10^5^ and a maximum fill time of 50 ms, using the stepped NCE of 25 and 35 for fragmentation in the HCD cell. Data were acquired from 50 to 1500 Da with dd-MS^2^ or MS^2^ in centroid mode. Raw data were acquired and processed using the Xcalibur software (Version 2.3.1, Thermo Electron Corporation, San Jose, CA, USA). Optimum separation of hericenones, hericenes, ergosterol and Erinacine A was achieved using an UHPLC column (Acquity HSS T3, 100 mm × 2.1 mm, 1.8 μm) from Waters (Milford, MA, USA).

### 3.3. Chromatographic Condition

The chromatographic separation was achieved under a gradient separation at 30 °C. Gradient elution was performed using 0.1% FA in water (solvent A) and acetonitrile (solvent B) with the gradient program listed in Table 1. The injection volume for UHPLC was 10 μL.

### 3.4. Standard Preparation

Individual standard stock solutions of 1~5 mg/mL of each *H. erinaceus* standard compound were accurately prepared by weighing about 5~20 mg of each compound and dissolving them into a 10 mL volumetric flask using ethanol. Volumetric flasks were sonicated for 10 min and wrapped with aluminum foil to protect them from light. Stock solutions were kept refrigerated. Working standard solutions were prepared fresh daily by pipetting aliquots of stock solutions and serial dilutions with ethanol were made at concentrations ranging from 2 µg/mL to 500 µg/mL for the standards of hericenones, hericenes, ergosterol and erinacine A.

### 3.5. Test Materials and Sample Preparation

For the sample preparation, 1×, 2× and 3× extractions were performed on one of the products. Approximately 500 mg of *H. erinaceus* fruiting body product was extracted with 10 mL, 5.0 mL or 3.0 mL ethanol (EtOH) and sonicated for 30 min at ambient temperature in a Fisher Scientific FSGPD10 ultrasonic bath. The extract was then centrifuged for 10 min at 3000× *g*, and the supernatant was transferred to a 10 mL volumetric flask. As necessary, the residual solids were re-extracted once more with 5 mL or twice with 3.0 mL EtOH using the same procedure, and the combined supernatants were brought to volume (10 mL) with EtOH. With the best extract efficiency, 3× extractions were performed for the sample preparations of all raw materials or products. Extract samples were passed through a 3 mL syringe fitted with a 0.22 µm nylon filter (VWR) and collected into amber glass HPLC vials for LC analysis. Unless otherwise specified, all samples were analyzed in triplicate or greater.

### 3.6. Method Validation Parameters

Method validation was conducted in accordance with AOAC guidance and ICH Q2 recommendations for single-laboratory validation [22,23]. Individual reference standards were prepared as stock solutions (1–5 mg/mL) by dissolving each compound in ethanol (EtOH) in volumetric flasks. Stock solutions were stored at −20 °C for long-term stability. For calibration and system suitability, mixed working standard solutions were prepared by combining appropriate volumes of each stock to obtain target concentrations of 100–300 µg/mL for hericenones, hericenes, and ergosterol, and 50 µg/mL for erinacine A. These mixtures were subsequently diluted to appropriate concentrations to establish retention time and combined at different concentration levels for external calibration.

#### 3.6.1. Specificity/Resolution

The mixed reference standard was injected into the UHPLC-UV to establish the selectivity of the method. The resolution for each reference standard was calculated. An R_s_ > 1.5 between closely eluting components was considered acceptable for hericenones, hericenes, ergosterol and erinacine A.

#### 3.6.2. Linearity

The linearity for the reference standard was determined by a six- or five-point standard calibration curve. The standard curves for hericenones and hericenes ranged from 10 µg/mL to 300 µg/mL (10, 20, 38, 75, 150 and 300 µg/mL). The standard curve for ergosterol ranged from 7 µg/mL to 226 µg/mL (7, 14, 28.3, 56.5, 113 and 226 µg/mL). The standard curve for erinacine A ranged from 1.6 µg/mL to 51.4 µg/mL (1.6, 3.2, 6.4, 12.9, 25.7 and 51.4 µg/mL). The standard curve for Deacylhericenone and Deacylhericene ranged from 3 µg/mL to 100 µg/mL (3, 6, 12, 25, 50 and 100 µg/mL). A simple linear regression was used to calculate the R^2^ value, the slope, and the y-intercept of each curve for each analyte. An R^2^ ≥ 99.9% was considered acceptable. The calibration standards of hericenones, hericenes, ergosterol and erinacine A were triplicated at the five or six concentrations and analyzed over three days.

#### 3.6.3. LOD and LOQ

The limit of detection (LOD) and limit of quantification (LOQ) of the hericenones, hericenes, ergosterol and erinacine A standard assays were determined from the calibration curve method, according to ICH Q2 (R1) recommendations [23] by analyzing at least three replicates of the calibration standards. The LOD and LOQ of the proposed method were calculated using the following equation:

$$LOD=\frac{3.3\times Stdev\;y-intercept\;of\;Calibration\;Curve}{Slope\;of\;calibration\;curve\;(A_{ave})}$$

$$LOQ=\frac{10\times Stdev\;y-intercept\;of\;Calibration\;Curve}{Slope\;of\;calibration\;curve\;(A_{ave})}$$

#### 3.6.4. Recovery

Considering the cost and availability of reference standards, spike recovery experiments were performed at two levels (medium: 200 µg/g, low: 80 µg/g) for ergosterol. Powdered *H. erinaceus* material (after EtOH extraction) was analyzed for ergosterol before the standards were spiked. The appropriate amount of reference standards was used to spike the powdered *H. erinaceus* material, followed by the extraction process. Three replicates were performed at each level, and the mean recovery was calculated.

#### 3.6.5. Precision

Two independent replicates (Raw_01 and FP-01) of the same sample were prepared and analyzed on three separate days (n = 5 × 3). The within-day, between-day, and overall precision for all target compounds were calculated for single-laboratory validation.

## 4. Conclusions

In summary, a UHPLC–UV method was developed and single-laboratory validated for the simultaneous determination of major *H. erinaceus* marker compounds—hericenones, hericenes, erinacines, and ergosterol—in both raw materials and finished products derived from fruiting bodies and/or mycelium. Method validation followed AOAC guidance for dietary supplement and botanical methods, supporting fit-for-purpose use in routine quality control environments. The method provides reliable chromatographic separation and consistent marker quantitation across diverse product types, with a total run time of around 38 min under the optimized gradient conditions. The major hericenones, hericenes, ergosterol and erinacines were well-separated under the LC resolution. The SLMV data demonstrate acceptable performance of the presented (U)HPLC–UV-based method for quantitation of hericenones, hericenes, ergosterol and erinacines in *H. erinaceus* materials.

In conclusion, this SLMV method strengthens the analytical basis for characterizing *H. erinaceus* materials by enabling reliable, standardized marker measurements across product types. In the future, this method could be extended with more available hericenone, hericene and erinacine standard compounds. By improving batch-to-batch comparability, it enhances quality control and supports better standardization of materials for research and clinical studies while helping reduce variability among commercial products on the market.

## Figures and Tables

**Table 1:** Gradient UHPLC elution profile.

Time/min	A (0.1% FA, %)	B (ACN, %)	Flow Rate (mL/min)
0	100	0	0.39
3.86	90	10	
3.96	80	20	
5.98	60	40	
6.00	30	70	
6.30	20	80	
8.00	15	85	
8.90	15	85	0.39
8.95	15	85	0.5
10.90	10	90	
19.65	5	95	
20.10	0	100	
33.10	0	100	
33.20	100	0	
36.10	100	0	0.39
38.50	End		0.39

**Table 2:** Chromatographic parameters of hericenones, hericenes, ergosterol and erinacine.

Compound	Retention Factor (*k*′)	Relative RT (*α*)	Chromatographic Resolution (*R_s_*)
deacylhericenone (1)	9.31	-	-
erinacine A (2)	9.57	α_1/2_ = 1.026	R_1/2_ = 3.32
deacylhericene (3)	10.33	α_2/3_ = 1.072	R_2/3_ = 9.99
hericenone E (4)	20.65	α_3/4_ = 1.910	R_3/4_ = 67.54
ergosterol (5)	22.15	α_4/5_ = 1.069	R_4/5_ = 5.68
hericenone C (6)	23.61	α_5/6_ = 1.063	R_5/6_ = 4.99
hericenone D (7)	27.15	α_6/7_ = 1.144	R_6/7_ = 14.16
hericene D (8)	27.55	α_7/8_ = 1.014	R_7/8_ = 1.62
hericene A (9)	30.54	α_8/9_ = 1.105	R_8/9_ = 10.10
hericene C (10)	36.29	α_9/10_ = 1.182	R_9/10_ = 13.89

**Table 3:** Calibration parameters for hericenones, hericenes, ergosterol and erinacine from three different calibration curves.

	Calib Range (μg/mL)	Slope (±SD) ^a^	Y-Intercept (±SD) ^a^	r^2^ (±SD) ^a^	LOD (μg/mL)	LOQ (μg/mL)
DA ^b^-hericenone	2.83~90.40	63,903,100 ± 1,215,192.4	16,263.5 ± 6918.2	0.99996 ± 0.00002	0.325	1.083
DA-lhericene	3.08~98.40	54,773,000 ± 376,802.3	28,131.3 ±11,552.3	0.99991 ± 0.00008	0.633	2.109
Erinacine A	1.61~51.44	21,535,833 ± 782,834.0	3902.8 ± 1381.7	0.99993 ± 0.00004	0.192	0.642
Ergosterol	7.06~226.0	29,124,433 ± 151,712.6	62,057.0 ± 24,325.5	0.99920 ± 0.00019	2.506	8.352
Hericenone E	7.03~225.0	29,696,267 ± 287,794.8	25,623.1 ± 13,809.2	0.99996 ± 0.00004	1.395	4.650
Hericenone C	12.81~410.0	33,387,900 ± 318,969.0	44,721.3 ± 30,927.5	0.99998 ± 0.00002	2.779	9.263
Hericenone D	5.75~184.0	34,537,733 ± 576,568.4	33,612.8 ± 15,696.9	0.99991 ± 0.00006	1.363	4.545
Hericene D	13.94~446.0	34,715,100 ± 408,503.7	57,531.9 ± 37,227.3	0.99997 ± 0.00002	3.217	10.72
Hericene A	12.06~386.0	29,943,967 ± 332,375.6	55,499.7 ± 5551.5	0.99993 ± 0.00006	0.556	1.854
Hericene C	10.38~332.0	33,280,800 ± 396,768.5	48,234.3 ± 37,197.8	0.99985 ± 0.00006	3.353	11.18

Note: Calibration curves were performed three times on different days and established by measuring the concentration vs. the corresponding peak area. ^a^ The given values are the mean of three replicates ± standard deviation. ^b^ DA = Deacyl.

**Table 4:** Spike recovery results of UHPLC-UV method for determination of ergosterol.

	Ergosterol	
	0.2%	0.08%
Native (μg/mL) ^a^	1.75 ± 0.31	1.75 ± 0.31
Spiked (μg/mL) ^b^	104.43	41.77
After Spiked (μg/mL) ^b^	106.18 ± 0.31	43.52 ± 0.31
Detected (μg/mL) ^a^	98.38 ± 1.26	42.50 ± 2.02
Marginal recovery (%) ^a^	92.531 ± 1.21	97.57 ± 4.83
Total Recovery (%) ^a^	92.65 ± 1.19	97.67 ± 4.63

Note: ^a^ The given values are the mean of three replicated measurements ± standard deviation. ^b^ The values for all the spiked and after spiked concentrations were calculated as the mean of three replicates plus the standard deviation.

**Table 5:** Precision summary of UHPLC-UV method for detecting hericenones, hericenes, ergosterol and erinacine in *H. erinaceus* products.

		DA-Hericenone		Erinacine A	DA-Hericene		Ergosterol	Hericenone			Hericene		
								E	C	D	D	A	C
		MFP-01											
LOQ (mg/g)		0.0217		0.0128	0.0422		0.1671	0.093	0.1853	0.0909	0.2145	0.0371	0.2235
D1	Mean ± SD (mg/g)	0.0157 ± 0.0008		0.238 ± 0.0003	BLQ		BLQ	BLQ	BLQ	BLQ	BLQ	BLQ	BLQ
	RSD_r_ (%)	4.99		1.26	BLQ		BLQ	BLQ	BLQ	BLQ	BLQ	BLQ	BLQ
D2	Mean ± SD (mg/g)	0.0152 ± 0.0009		0.242 ± 0.005	BLQ		BLQ	BLQ	BLQ	BLQ	BLQ	BLQ	BLQ
	RSD_r_ (%)	5.99		2.16	BLQ		BLQ	BLQ	BLQ	BLQ	BLQ	BLQ	BLQ
D3	Mean ± SD (mg/g)	0.0169 ± 0.0015		0.251 ± 0.003	BLQ		BLQ	BLQ	BLQ	BLQ	BLQ	BLQ	BLQ
	RSD_r_ (%)	8.83		1.24	BLQ		BLQ	BLQ	BLQ	BLQ	BLQ	BLQ	BLQ
Mean ± SD (mg/g)		0.0160 ± 0.0009		0.244 ± 0.006	BLQ		BLQ	BLQ	BLQ	BLQ	BLQ	BLQ	BLQ
RSD (%) Interday		5.72		2.65	BLQ		BLQ	BLQ	BLQ	BLQ	BLQ	BLQ	BLQ
		**Raw-01**											
D1	Mean ± SD (mg/g)	0.0374 ± 0.00031	ND			0.1335 ± 0.00362	1.656 ± 0.0209	0.033 ± 0.0011	0.1374 ± 0.0038	0.063 ± 0.0016	0.0545 ± 0.00115	0.256 ± 0.0087	0.100 ± 0.0028
	RSD_r_ (%)	0.83	ND			2.71	1.26	3.24	2.73	2.45	2.12	3.39	2.74
D2	Mean± SD (mg/g)	0.0346 ± 0.00083	ND			0.1292 ± 0.00586	1.630 ± 0.0510	0.031 ± 0.00112	0.133 ± 0.0055	0.061 ± 0.0024	0.050 ± 0.0016	0.246 ± 0.0061	0.098 ± 0.0029
	RSD_r_ (%)	2.4	ND			4.54	3.13	3.57	4.15	3.9	3.2	2.46	2.98
D3	Mean± SD (mg/g)	0.0357 ± 0.00068	ND			0.1304 ± 0.00095	1.666 ± 0.0416	0.0307 ± 0.0011	0.134 ± 0.0041	0.061 ± 0.0022	0.054 ± 0.0013	0.253 ± 0.0085	0.102 ± 0.0029
	RSD_r_ (%)	1.9	ND			0.73	2.49	3.42	3.02	3.62	2.44	3.36	2.89
Mean ± SD (mg/g)		0.036 ± 0.00137	ND			0.131 ± 0.0022	1.651 ± 0.0183	0.032 ± 0.00099	0.135 ± 0.0022	0.062 ± 0.0012	0.053 ± 0.0024	0.252 ± 0.0050	0.100 ± 0.0022
RSD_r_ (%) Interday		3.83	ND			1.71	1.11	3.13	1.6	1.97	4.61	1.98	2.22

Note: D-1/2/3 are day-1/2/3; MFP: mycelia finished product; Raw-01: fruit body raw material; ND: not detectable; BLQ: below LOQ; All RSD_r_ are intraday for each day.

**Table 6:** Summarized quantitative results of hericenones, hericenes, ergosterol and erinacine A in *H. erinaceus* raw materials and finished products measured by the method of UHPLC–UV.

		DA-Hericenone	DA-Hericene	Ergosterol	Hericenone			Hericene		
					E	C	D	D	A	C
LOQ (mg/g)		0.0217	0.0422	0.1671	0.093	0.1853	0.0909	0.2145	0.0371	0.2235
Raw_02	Mean ± SD (mg/g)	0.0337 ± 0.0014	0.0374 ± 0.0017	0.826 ± 0.024	0.0289 ± 0.0017	0.1071 ± 0.0036	0.0586 ± 0.0006	0.0534 ± 0.0019	0.116 ± 0.0053	0.0519 ± 0.0010
	RSD_r_ (%)	4.07	4.62	2.9	5.96	3.32	0.99	3.54	4.59	1.86
Raw_3	Mean ± SD (mg/g)	0.0193 ± 0.00045	0.0191 ± 0.00087	1.035 ± 0.0176	0.0550 ± 0.00048	0.3439 ± 0.0086	0.0292 ± 0.0023	0.0829 ± 0.0058	0.2363 ± 0.0117	0.0967 ± 0.0046
	RSD_r_ (%)	2.34	4.56	1.7	0.88	2.51	7.98	7.05	4.95	4.8
FFP_1	Mean ± SD (mg/g)	0.0490 ± 0.0005	0.1272 ± 0.0006	1.938 ± 0.0279	0.0204 ± 0.0011	0.0374 ± 0.0009	0.0215 ± 0.0006	0.0993 ± 0.0006	0.3186 ± 0.0041	0.0967 ± 0.0046
	RSD_r_ (%)	1.08	0.5	1.44	5.57	2.33	2.78	0.64	1.28	1.4
FFP_2	Mean ± SD (mg/g)	0.1002 ± 0.0006	0.1351 ± 0.0033	1.866 ± 0.006	0.1088 ± 0.0043	0.5030 ± 0.0065	0.2417 ± 0.0073	0.1480 ± 0.0043	0.8162 ± 0.0089	0.3272 ± 0.0031
	RSD_r_ (%)	0.57	2.43	0.3	3.91	1.29	3.03	2.9	1.09	0.96
FFP_3	Mean ± SD (mg/g)	0.0350 ± 0.0002	0.0721 ± 0.0012	1.200 ± 0.006	0.0836 ± 0.0017	0.0988 ± 0.0040	0.0571 ± 0.0009	0.1012 ± 0.0036	0.5417 ± 0.0060	0.2376 ± 0.0029
	RSD_r_ (%)	0.45	1.63	0.5	2.07	4.05	1.53	3.57	1.12	1.23
FFP_4	Mean ± SD (mg/g)	0.0245 ± 0.0023	0.1031 ± 0.0057	0.5202 ± 0.0379	0.0100 ± 0.0012	0.00481 ± 0.0004	0.00553 ± 0.0004	0.0443 ± 0.0032	0.0840 ± 0.0077	0.0347 ± 0.0023
	RSD_r_ (%)	9.53	5.56	7.28	12.14	8.34	6.56	7.28	9.13	6.6
FFP_5	Mean ± SD (mg/g)	0.0237 ± 0.0004	0.0362 ± 0.0003	1.798 ± 0.0179	0.1851 ± 0.0031	0.241 ± 0.0065	0.1451 ± 0.0010	0.398 ± 0.0033	0.985 ± 0.0043	0.349 ± 0.0147
	RSD_r_ (%)	1.78	0.7	1	1.7	2.70	0.71	0.83	0.44	4.21
FFP_6	Mean ± SD (mg/g)	ND	ND	0.6756 ± 0.016	0.0738 ± 0.0022	0.2664 ± 0.0027	0.0885 ± 0.0038	0.0632 ± 0.0019	0.4125 ± 0.0027	0.110 ± 0.0020
	RSD_r_ (%)	ND	ND	2.29	2.94	1.01	4.28	2.98	0.66	1.83
FFP_7	Mean ± SD (mg/g)	0.0243 ± 0.0004	0.0145 ± 0.0004	1.418 ± 0.0056	0.1098 ± 0.0042	0.2944 ± 0.0076	0.1001 ± 0.0054	0.1067 ± 0.0042	0.6003 ± 0.0024	0.158 ± 0.0045
	RSD_r_ (%)	1.82	2.92	0.4	3.85	2.6	5.34	3.95	0.39	2.81

Note: Raw-2/3 are fruit body raw materials; FFP: fruit body finished product; ND: not detectable; All RSD_r_ are intraday here.

## Data Availability

The original contributions presented in this study are included in the article/Supplementary Materials. Further inquiries can be directed to the corresponding authors.

## Supplementary Materials

The following supporting information can be downloaded at: https://www.mdpi.com/article/10.3390/molecules31030569/s1, Figure S1: UV spectra of major hericenones, hericenes, ergosterol and erinacine A; Figure S2: Mass spectra of major hericenones, hericenes and erinacine A; Figure S3: Represented UHPLC-UVs for *H. erinaceus* raw material for the method development; Figure S4: Calibration curves of major hericenones, hericenes, ergosterol and erinacine A; Figure S5: UHPLC-UV screening of major hericenones, hericenes, ergosterol and erinacine A in some *H. erinaceus* products; Table S1: Extraction efficiency tests (1×, 2× and 3× extractions to final vol 10 mL) on Finished Product 5; both 10 mL × 1 and 5 mL × 2 were compared to 3 mL × 3 for the relative extraction yields.
