# Supplementary material for: Simultaneous UHPLC-UV Determination of Hericenones, Hericenes, Erinacines and Ergosterol in Hericium erinaceus Raw Materials or Products
Source: Molecules. 2026 Feb 6;31(3):569. doi: 10.3390/molecules31030569 (PMC12899107; doi:10.3390/molecules31030569)
Supplement: Supplementary file 1 [file molecules-31-00569-s001.zip › molecules-4110653-supplementary.pdf]

Title: Simultaneous UHPLC-UV Determination of Hericenones, Hericenes, Erinacines and Ergosterol in *Hericium erinaceus* Raw Materials or Products

Yijin Tang 1 *, Ozan Kahraman1, Anthony J. Goos1, and Christine Fields 1 *

[1] Applied Food Sciences, Inc., 2500 Crosspark Road, Coralville, IA 52241.

* Correspondence: ytang@appliedfoods.com; cfields@appliedfoods.com; Tel.: (+1-319-467-4502)

Keywords: *Hericium erinaceus* (*H. erinaceus*); UHPLC-UV; Hericenone; Hericene; Erinacine; Ergosterol; Single-Laboratory Method Validation (SLMV); Chemical Markers; Quality control

Support Figure Legend

Figure S1.	UV spectra of major hericenones, hericenes, ergosterol and erinacine A.
Figure S2.	Mass spectra of major hericenones, hericenes and erinacine A.
Figure S3.	Represented UHPLC-UVs for H. erinaceus raw material for the method development.
Figure S4.	Calibration curves of major hericenones, hericenes, ergosterol and erinacine A.
Table S1.	Extraction efficiency tests (1x, 2x and 3x extractions to final vol 10ml) on Finished Product 5; both 10ml x1 and 5ml x 2 were compared to 3ml x 3 for the relative extraction yields.
Figure S5.	UHPLC-UV screening of major hericenones, hericenes, ergosterol and erinacine A in some H. erinaceus products.

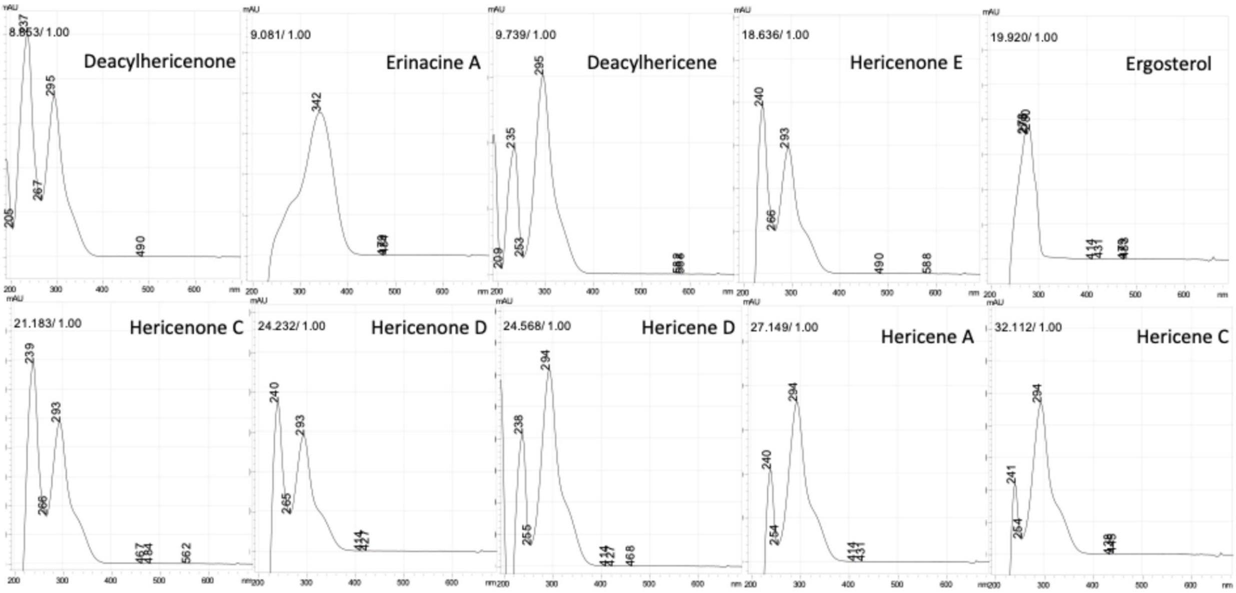
8.853/ 1.00
Deacylhericenone
9.081/ 1.00
Erinacine A
9.739/ 1.00
Deacylhericene
18.636/ 1.00
Hericenone E
19.920/ 1.00
Ergosterol
21.183/ 1.00
Hericenone C
24.232/ 1.00
Hericenone D
24.568/ 1.00
Hericene D
27.149/ 1.00
Hericene A
32.112/ 1.00
Hericene C

Figure S1

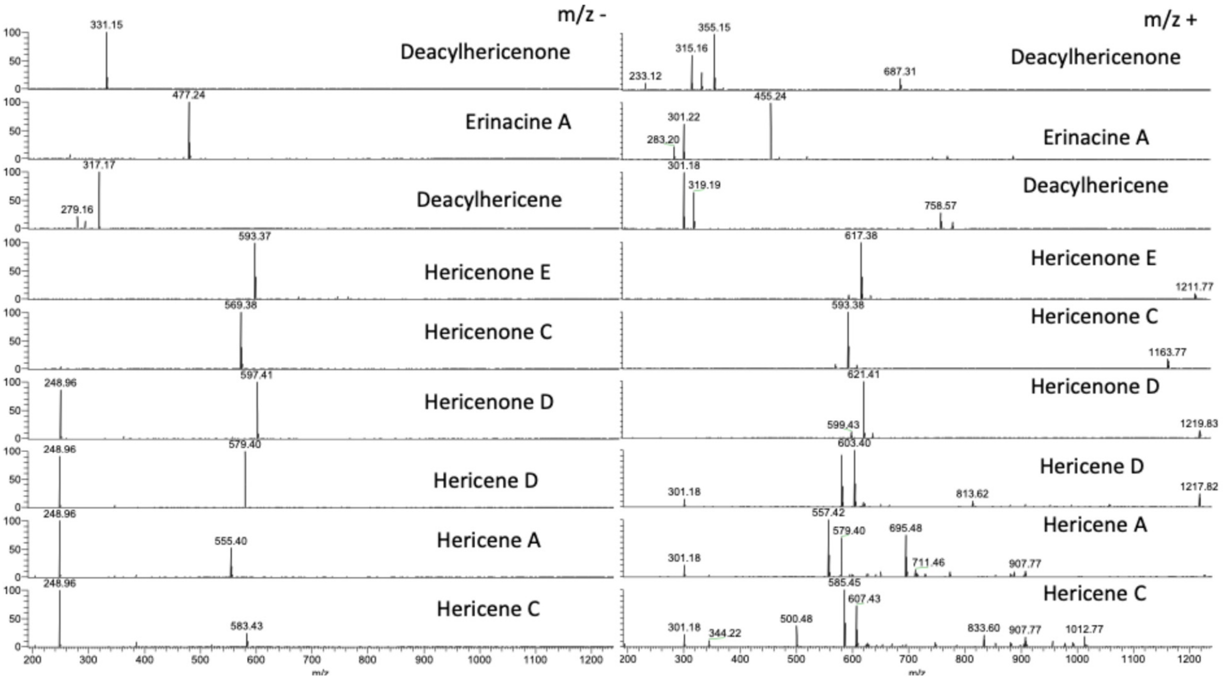
m/z -
m/z +
Deacylhericenone
331.15
355.15
315.16
233.12
687.31
Erinacine A
477.24
455.24
301.22
283.20
Deacylhericene
317.17
279.16
301.18
319.19
758.57
Hericenone E
593.37
617.38
1211.77
Hericenone C
569.38
593.38
1163.77
Hericenone D
248.96
597.41
621.41
599.43
1219.83
Hericene D
248.96
579.40
603.40
301.18
813.62
1217.82
Hericene A
248.96
555.40
557.42
579.40
695.48
711.46
301.18
907.77
Hericene C
248.96
583.43
585.45
607.43
301.18
344.22
500.48
833.60
907.77
1012.77
100
50
0
200 300 400 500 600 700 800 900 1000 1100 1200
m/z

Figure S2

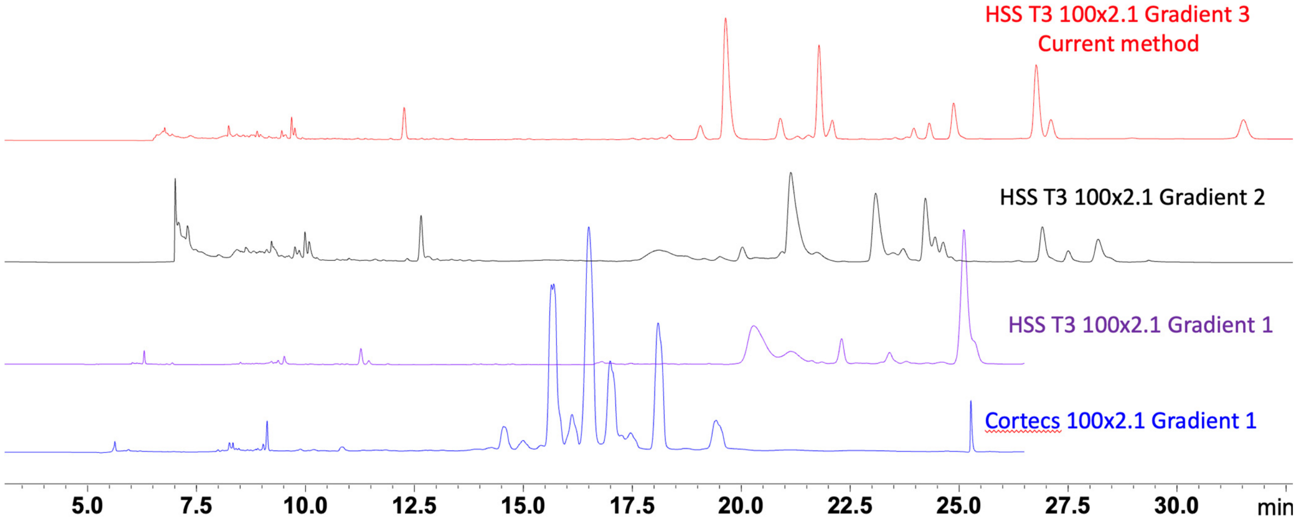
HSS T3 100x2.1 Gradient 3
Current method
HSS T3 100x2.1 Gradient 2
HSS T3 100x2.1 Gradient 1
Cortecs 100x2.1 Gradient 1
5.0
7.5
10.0
12.5
15.0
17.5
20.0
22.5
25.0
27.5
30.0
min

Figure S3

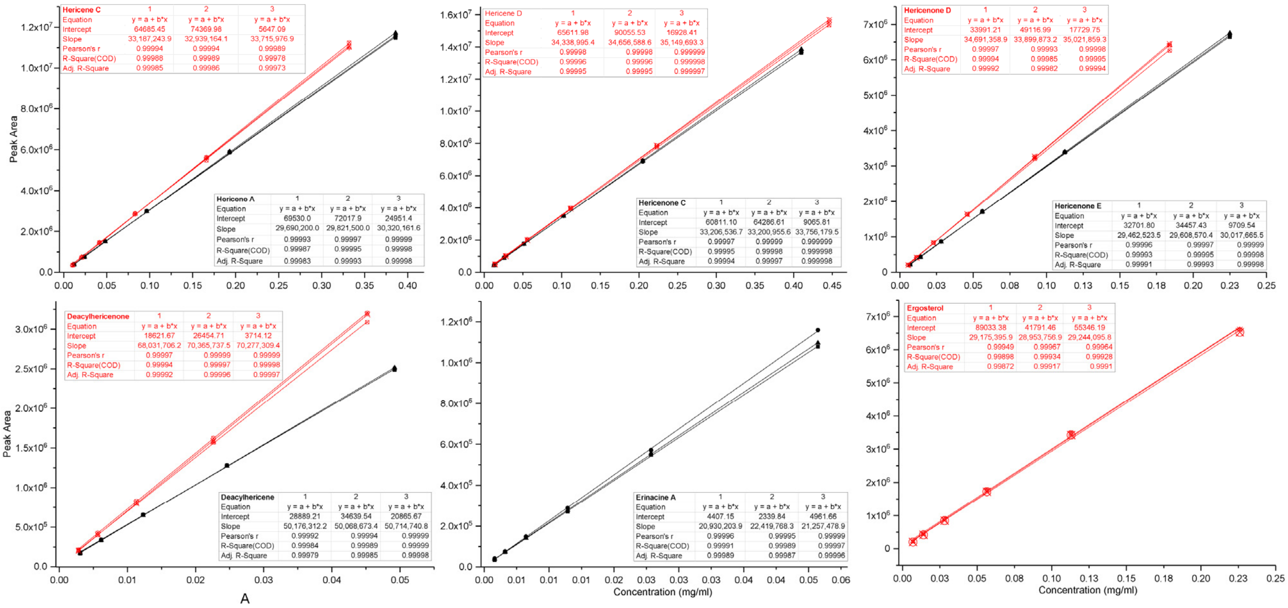
Hericene C 1 2 3
Equation y = a + b*x y = a + b*x y = a + b*x
Intercept 64685.45 74369.98 5647.09
Slope 33,187,243.9 32,939,164.1 33,715,976.9
Pearson's r 0.99994 0.99994 0.99989
R-Square(COD) 0.99988 0.99989 0.99978
Adj. R-Square 0.99985 0.99986 0.99973
Hericene A 1 2 3
Equation y = a + b*x y = a + b*x y = a + b*x
Intercept 69530.0 72017.9 24951.4
Slope 29,690,200.0 29,821,500.0 30,320,161.6
Pearson's r 0.99993 0.99997 0.99999
R-Square(COD) 0.99987 0.99995 0.99998
Adj. R-Square 0.99983 0.99993 0.99998
Hericene D 1 2 3
Equation y = a + b*x y = a + b*x y = a + b*x
Intercept 65611.98 90055.53 16928.41
Slope 34,338,995.4 34,656,588.6 35,149,693.3
Pearson's r 0.99998 0.99998 0.999999
R-Square(COD) 0.99996 0.99996 0.999998
Adj. R-Square 0.99995 0.99995 0.999997
Hericenone C 1 2 3
Equation y = a + b*x y = a + b*x y = a + b*x
Intercept 60811.10 64286.61 9065.81
Slope 33,206,536.7 33,200,955.6 33,756,179.5
Pearson's r 0.99997 0.99999 0.999999
R-Square(COD) 0.99995 0.99998 0.999998
Adj. R-Square 0.99994 0.99997 0.999998
Hericenone D 1 2 3
Equation y = a + b*x y = a + b*x y = a + b*x
Intercept 33991.21 49116.99 17729.75
Slope 34,691,358.9 33,899,873.2 35,021,859.3
Pearson's r 0.99997 0.99993 0.99998
R-Square(COD) 0.99994 0.99985 0.99995
Adj. R-Square 0.99992 0.99982 0.99994
Hericenone E 1 2 3
Equation y = a + b*x y = a + b*x y = a + b*x
Intercept 32701.80 34457.43 9709.54
Slope 29,462,523.5 29,608,570.4 30,017,665.5
Pearson's r 0.99996 0.99997 0.99999
R-Square(COD) 0.99993 0.99995 0.99998
Adj. R-Square 0.99991 0.99993 0.99998
Deacylhericenone 1 2 3
Equation y = a + b*x y = a + b*x y = a + b*x
Intercept 18621.67 26454.71 3714.12
Slope 68,031,706.2 70,365,737.5 70,277,309.4
Pearson's r 0.99997 0.99999 0.99999
R-Square(COD) 0.99994 0.99997 0.99998
Adj. R-Square 0.99992 0.99996 0.99997
Deacylhericene 1 2 3
Equation y = a + b*x y = a + b*x y = a + b*x
Intercept 28889.21 34639.54 20865.67
Slope 50,176,312.2 50,068,673.4 50,714,740.8
Pearson's r 0.99992 0.99994 0.99999
R-Square(COD) 0.99984 0.99989 0.99999
Adj. R-Square 0.99979 0.99985 0.99998
Erinacine A 1 2 3
Equation y = a + b*x y = a + b*x y = a + b*x
Intercept 4407.15 2339.84 4961.66
Slope 20,930,203.9 22,419,768.3 21,257,478.9
Pearson's r 0.99996 0.99995 0.99999
R-Square(COD) 0.99991 0.99989 0.99997
Adj. R-Square 0.99989 0.99987 0.99996
Ergosterol 1 2 3
Equation y = a + b*x y = a + b*x y = a + b*x
Intercept 89033.38 41791.46 55346.19
Slope 29,175,395.9 28,953,756.9 29,244,095.8
Pearson's r 0.99949 0.99967 0.99964
R-Square(COD) 0.99898 0.99934 0.99928
Adj. R-Square 0.99872 0.99917 0.9991
Peak Area
Peak Area
A
Concentration (mg/ml)
Concentration (mg/ml)

Figure S4

Name	mg/g	10ml x 1 (%)	5ml x 2 (%)
DeacylHericene	0.0237±0.0004	94.04	103.5
DeacylHericenone	0.0362±0.0003	94.90	98.53
Hericenone E	0.1851±0.0031	92.78	94.18
**Ergosterol**	**1.798±0.0179**	**87.61**	**91.48**
Hericenone C	0.241±0.0065	97.72	98.37
Hericenone D	0.1451±0.0010	99.76	101.2
Hericene D	0.398±0.0033	97.88	101.5
**Hericene A**	**0.985±0.0043**	**92.46**	**96.51**
Hericene C	0.349±0.0147	98.39	100.09

Table S1

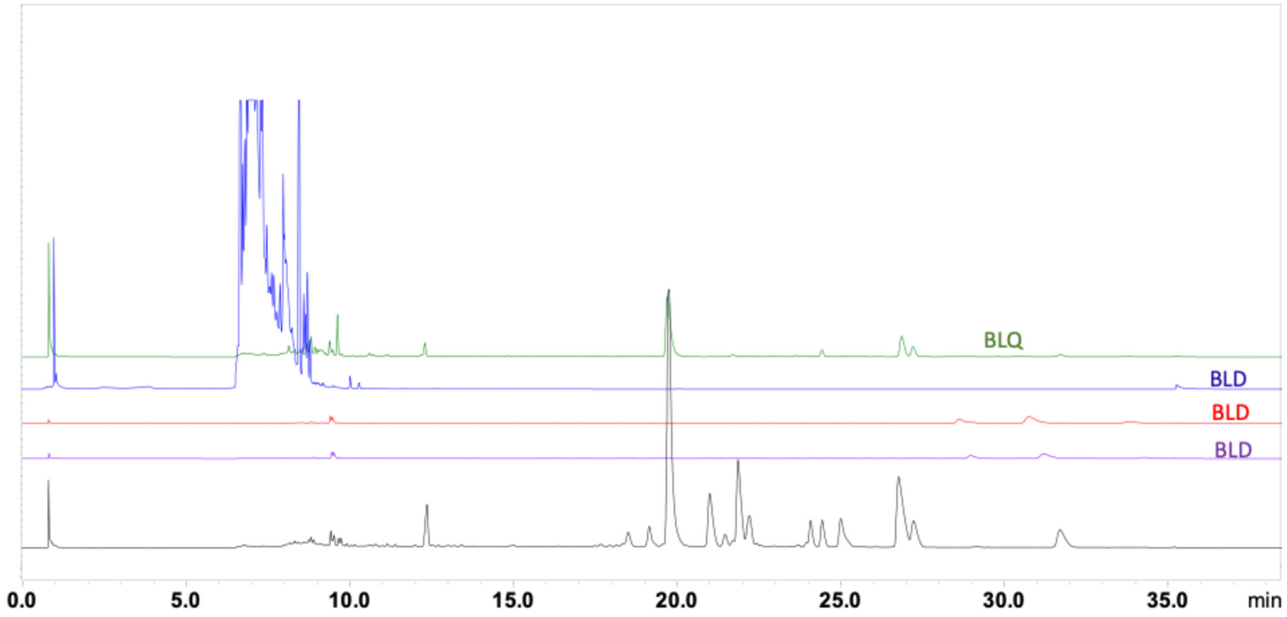

Figure S5
